# Bridging science and hope: the evolving story of gene therapy for neuromuscular diseases

**DOI:** 10.3389/fcell.2026.1765367

**Published:** 2026-04-14

**Authors:** Nicolas Wein, Florian Barthélémy

**Affiliations:** 1 Nantes Université, CHU de Nantes, INSERM, TaRGeT - Translational Research in Gene Therapy, UMR 1089, Nantes, France; 2 Center for Duchenne Muscular Dystrophy at UCLA, Los Angeles, CA, United States; 3 Department of Microbiology, Immunology and Molecular Genetics, David Geffen School of Medicine, College of Letters and Sciences, University of California, Los Angeles, Los Angeles, CA, United States

**Keywords:** exon skipping, gene editing, gene therapy, neuromuscular disease, RNA interference, personalized medicine

## Abstract

The field of gene therapy for neuromuscular dystrophies has evolved over the past two decades. Despite some outstanding positive outcomes, some unfortunate adverse effects also led to big setbacks. One important key point is to study relevant preclinical models and to embrace diverse strategies to mitigate or avoid such negative outcomes. Although at first, for some diseases, the promise of a one-treatment-for-all approach was envisioned, it has recently become clear that a personalized approach will likely be preferable given the high variability in response between individuals.

## Beyond potential: gene therapy’s breakthrough success in neuromuscular diseases

**FIGURE 1:**
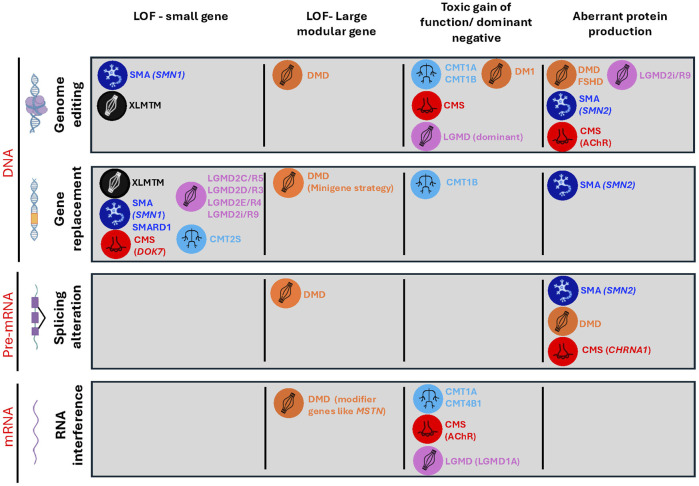
Mechanism-strategy matrix for therapeutic intervention. This matrix illustrates the alignment between therapeutic modalities and their respective molecular targets, categorized by the functional consequences of pathogenic mutations. The horizontal axis delineates the different modalities [gene replacement, splicing modulation, RNA interference (RNAi), and genome editing]. Primary molecular targets (DNA, pre-mRNA, and mRNA) for each are also indicated to the left. The vertical axis represents the pathophysiological classification according to their primary mutational mechanism: loss-of-function (LoF), toxic gain-of-function/dominant-negative, or aberrant protein production. Graphical icons denote the primary affected location for each disorder, including the muscle fibers (XLMTM, LGMD, DMD, DM1, and FSHD), the neuromuscular junction (CMS), and the peripheral nerve (CMT) or motor neuron (SMA). SMA, spinal muscular dystrophy; DMD, Duchenne muscular dystrophy; CMT, Charcot–Marie–Tooth; CMS, congenital myasthenic syndrome; LGMD, limb girdle muscular dystrophy; FSHD, facioscapulohumeral muscular dystrophy; DM1, myotonic dystrophy type 1; XLMTM, X-linked myotubular myopathy.

**FIGURE 2:**
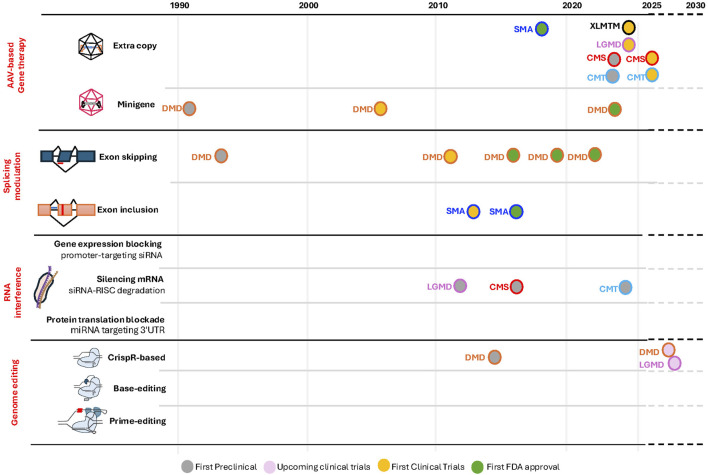
Chronological evolution of therapeutic modalities for representative neuromuscular disorders. The timeline illustrates key developmental milestones for the most frequently investigated diseases. Gray circles: initial preclinical proof-of-concept studies demonstrating therapeutic efficacy. Pink circles: imminent or planned first-in-class clinical investigations. Orange circles: initiation of first-in-human clinical trial enrollment. Green circles: initial regulatory approval by the FDA. Individual therapeutic modalities are outlined with color-coding corresponding to a specific disease, as defined in the legend. SMA, spinal muscular dystrophy; DMD, Duchenne muscular dystrophy; CMT, Charcot–Marie–Tooth; CMS, congenital myasthenic syndrome; LGMD, limb girdle muscular dystrophy; XLMTM, X-linked myotubular myopathy.

With the discovery of the correlation between genetic defects and diseases, the possibility of correcting these abnormalities has always been a driver of scientific discoveries and opened a new field of research: gene therapy. Genetic modulation of diseases, particularly the excision of defective portions of the genetic material, was first conceptualized in the late 1970s (Zamecnik and Stephenson, 1978). This technique relies on the use of DNA/RNA molecules (antisense oligonucleotides or AONs) to restore the reading frame by removing (“skipping”) one or more exons during pre-mRNA processing. This technique is particularly suitable for very large, modular proteins tolerant to internal deletions such as dystrophin in Duchenne muscular dystrophy (DMD) (See Figure 1). Based on the fact that patients presenting a milder form of the disease, Becker muscular dystrophy, have a missing dispensable part of dystrophin, leading to mild symptoms, several groups developed exon-skipping approaches using AONs, allowing the removal of the portion carrying the mutation from the mRNA while maintaining the open reading frame (Takeshima et al., 1995; Pramono et al., 1996; Gebski et al., 2003). In the recent years, multiple AONs have been approved by the FDA notably for DMD (eteplirsen for exon 51, golodirsen and viltolarsen for exon 53, and casimersen for exon 45) despite relatively modest efficacy (between 0.3% and 6% of native dystrophin protein levels were reached in treated patients) (Aartsma-Rus and Krieg, 2017; Dhillon, 2020; Heo, 2020; Shirley, 2021) (see Figure 2). Although the personalized exon-skipping strategy allows partial restoration of the protein, the design, efficacy, and off-target screening must be conducted for each exon. A new generation of exon-skipping technologies relying on chemical backbone modifications, peptide conjugation, antibody conjugation (to guide oligonucleotides to specific tissues via endocytosis of highly expressed receptors), or viral delivery of U7 snRNA-based therapies (that recognize selected splice sites or exonic splicing enhancers in the target pre-mRNA, thereby blocking normal spliceosome assembly and forcing its excision from the mature mRNA) (Odermatt et al., 2016; Gushchina et al., 2023; McDowall et al., 2024; Krey-Grauert et al., 2025; Wein et al., 2017) is also being investigated in clinical trials, and some, such as Del-Zota (Avidity Biosciences), which uses an antibody-conjugated AON to target *DMD* exon 44, are expected to undergo FDA review in 2026 (Avidity Biosciences Announces, 2025) (Figure 2). Exon-skipping strategies could also be employed for congenital myasthenic syndrome (CMS) due to *CHRNA1* aberrant exon P3A inclusion; excision of the nonfunctional exon corrects splicing *in vitro*, showing that AON-mediated exon skipping can normalize neuromuscular junction (NMJ) receptor composition (Tei et al., 2015). A similar splicing modulation, also relying on the use of AON, promotes the inclusion of an exon carrying a splicing-causing variant [such as *SMN2* exon 7 for spinal muscular atrophy (SMA)], allowing the expression of a near-normal functional protein (Singh et al., 2009). Based on very impressive results in clinical trials, especially in the infant form of the disease (SMA type 1) (Finkel et al., 2017), nusinersen/Spinraza was approved in December 2016 by the FDA (Aartsma-Rus, 2017) (Figure 2).

Although those exon-skipping/inclusion approaches seemed very promising, the field also investigated other more universal approaches, which would be applicable to most patients. In the case of DMD, the discovery of a truncated dystrophin, lacking 70% of the native protein sequence but partially functional, in a 61-year-old ambulant patient was a game changer (England et al., 1990). This proved that removing potentially nonessential parts of the dystrophin retains partial functions. Moreover, the use of adeno-associated virus (AAV) to deliver a smaller cDNA encoding for micro-dystrophin represents a largely mutation-independent approach and could benefit nearly all patients with DMD (Duan, 2018; Wang et al., 2019a). To deliver this mini-gene, the field relies on the use of AAV, which, despite a very limited encapsulation limit (4.7 kb), remains relatively safe at low-to-medium doses. Elevidys (delandistrogene moxeparvovec-rokl), an AAV-based delivery of a mini-dystrophin gene, received a controversial FDA approval for DMD in 2023 (see Figure 2). Indeed, all pivotal trials repeatedly failed to show statistically significant protein expression levels posttreatment, which was correlated with no gains in motor outcomes or strength compared to placebo (Hoy, 2023). This approach is also suitable for small genes with a higher frequency of recessive loss of function [e.g., *SMN1* in SMA (Mendell et al., 2017), *SGCA* in limb girdle muscular dystrophy (LGMD2D/R3) (Mendell et al., 2010), or *FKRP* (LGMD2i/R9) (Gicquel et al., 2017) which has beginning enrolling for a phase I clinical trial (NCT05224505), Charcot–Marie–Tooth (CMT) 1B linked to *MPZ* mutations with progress recently made public (Munezero et al., 2024) and several CMS-associated genes such as *DOK7* (Cossins et al., 2025)] that could also be eligible (Figure 1).

The delivery of a full copy of *SMN1* for SMA patients, who do not express this gene, using an AAV-based delivery (Zolgensma (onasemnogene abeparvovec-xioi), was astonishing and became the first FDA-approved gene therapy for a neuromuscular disorder in 2019 (see Figure 2) with all 29 pre-symptomatically treated children achieving age-appropriate milestones (Hoy, 2019). Based on follow-up studies and real-life outcomes, the treatment was fully approved to all patients aged 2 and older in November 2025 (FDA, 2025) (Figure 2).

Other promising gene therapies are being successfully tested in clinical trials and notably for LGMD or CMS (Wu et al., 2025; Mendonca and Zanoteli, 2022). Sarepta’s bidridistrogene xeboparvovec (SRP-9003) showed promising results in phase 1/2 trials for LGMD2E/R4 linked to β-sarcoglycan mutations, achieving robust protein expression (62.1% of normal in the higher dose cohort) 2 years post-injection. This was associated with motor improvements for 3 years posttreatment (Mendell et al., 2024a). This drug is now in a phase 3 clinical trial (EMERGENE/NCT06246513), with expected results by the end of 2025. Sarepta has also initiated and discontinued trials for its candidate drugs SRP-9005-101, a single-dose AAV vector carrying the human γ-sarcoglycan transgene for LGMD2C/R5 (NCT06952686), and SRP-9004-102, an AAV-based gene therapy delivering a copy of α-sarcoglycan for LGMD2D/R3 (NCT06747273) (Figures 1, 2), due to strategic decisions.

In parallel, several approaches are being evaluated for other diseases using AAV and intrathecal injections (Nair et al., 2023) (Figure 1). For example, an IGHMBP2 gene replacement therapy for *IGHMBP2*-related diseases, specifically SMA with respiratory distress type 1 (SMARD1) and Charcot–Marie–Tooth disease type 2S (CMT2S), is currently underway. SMARD1 is a rare autosomal recessive motor neuron disorder presenting in infancy, characterized by early respiratory failure due to diaphragmatic paralysis, progressive distal muscle weakness, and sensory neuropathy, whereas CMT2S, caused by biallelic mutations, leads to progressive distal muscle weakness and sensory loss. This approach shows significant improvement in the neuromuscular pathology, together with an improvement in survival, motor performance, compound muscle action potential (CMAP), and motor unit number estimation (MUNE) across the three IGHMBP2 mouse models covering the SMARD1–CMT2S spectrum (McCulloch et al., 2024). This approach led to a phase I/IIa clinical trial (NCT05152823) currently enrolling (Figure 2).

Finally, an AAVrh74 vector delivering *DOK7* under a muscle-restricted promoter, administered intraperitoneally, is being tested for CMS in a phase Ib trial (NCT06436742) after it demonstrated enlarged neuro-muscular junctions, restoration of strength to at least wild-type levels, and robust DOK7 expression in the diaphragm and tibialis anterior in animal models (Cossins et al., 2025).

In addition to exon-skipping and gene-transfer strategies, RNA interference (RNAi) is a promising approach that uses small RNA molecules (such as siRNA or miRNA) to guide protein complexes to target and silence specific messenger RNA (mRNA), thereby blocking gene expression by degrading the mRNA or preventing its translation, making this approach particularly suitable for dosage-sensitive or dominant-negative disorders, including CMT1A (Stavrou et al., 2022) and CMT1B (Kolesnik et al., 2024), dominant forms of LGMD such as LGMD1A (Liu et al., 2012), slow-channel CMS linked to AChR mutations (Abdelgany et al., 2003), and disease modifier genes such as *MSTN* in DMD (Kim et al., 2016; Roberts et al., 2012) (Figures 1, 2). A single intrathecal injection of AAV9-miR871 targeting *PMP22* in CMT1A mouse models led to significant improvement in nerve conduction, motor function, and myelin structure, showing promises for a human trial (Stavrou et al., 2022). AAV9-mediated gene therapy delivering *MTMR2* to Schwann cells in a CMT4B1 mouse model successfully reduced myelin outfoldings and improved peripheral nerve pathology, demonstrating therapeutic potential for *MTMR2*-associated CMT4B1 (Vaccari et al., 2011).

However, these approaches, despite being promising, still have room for improvement in terms of efficacy, delivery, and safety (see next section). Most of these strategies still rely on the use of immunosuppression protocols to reduce side effects mainly by dampening the patient’s immune response to the viral vector and the newly expressed transgene (Foster et al., 2023).

Finally, a few innovative nonviral gene therapies (e.g., Engensis plasmid for human growth factor delivery) exhibit strong safety profiles and regenerative potential in neuropathy but are still in the very early phases of research development (Kessler et al., 2021).

## Promise meets reality: the scientific, clinical, and logistic hurdles in neuromuscular gene therapy

The field has recently experienced significant setbacks highlighting the ongoing challenges for both mini-gene transfer and exon-skipping therapeutic strategies. For example, some DMD gene therapy programs failed their pivotal trials: Sarepta’s EMBARK trial (NCT05096221) missed its primary endpoint despite positive secondary outcomes leading to the approval of delandistrogene moxeparvovec for patients aged 4–5 years by the FDA in 2023 (Hoy, 2023; Mendell et al., 2025). Results from Pfizer’s phase 3 CIFFREO trial (NCT04281485 for fordadistrogene movaparvovec) showed no improvement in the North Star Ambulatory Assessment and other secondary endpoints (time to rise and 10 m run/walk) in a cohort of ambulatory boys aged 4–7 years with DMD versus placebo, ending all development of their mini-dystrophin therapy program (Chwalen et al., 2025).

Exon-skipping drugs for DMD were also conditionally approved and required a long-term follow-up to prove efficacy and safety, but a recent setback came from the announcement that the nine-year-long ESSENCE trial (NCT04281485) did not show a statistically significant difference for Sarepta’s Vyondys 53 and Amondys 45 compared to the placebo on a key measurement of DMD patient mobility, thereby missing its primary endpoint (PPMD, 2025).

Another concern is the safety of these drugs. The liver toxicity of AAV has become clear with liver morbidity in the MTM1 trial (Shieh et al., 2020; Shieh et al., 2023). There were also three acute liver failures in patients who received a prospective therapeutic treatment using the AAVrh74 platform from Sarepta (two from patients treated with Elevidys and one from a patient enrolled in the SRP-9004 phase 1 trial for LGMD2D/R3). This led the FDA to put these trials on clinical holds (FDA News Release, 2025; Sarepta Therapeutics, 2025). The same happened to the Pfizer mini-dystrophin gene therapy approach due to a patient death in the phase 2 DAYLIGHT trial (NCT04281485) (Philippidis, 2024). Due to these setbacks, the field has been trying to find alternative strategies mainly by using immunosuppression protocols to avoid immune reaction to the virus (Kistner et al., 2024; Vrellaku et al., 2024; Wolff et al., 2025). The rituximab + sirolimus regimen can prevent the antibody spike and complement activation that leads to thrombotic microangiopathy (TMA) (Salabarria et al., 2024). Another alternative is the use of a triple combination of rituximab, sirolimus, and corticosteroids, which seem to prevent vector-related adverse events (Flotte et al., 2022). Finally, a prednisolone + rapamycin double therapy showed a sustained antibody suppression for up to 6 months in macaques (Kistner et al., 2024) but as yet to be tested in Humans. Given the association between complement activation and TMA, trials now monitor complement activation as an early indicator of severe immune reactions (Wolff et al., 2025).

The manufacturing cost, various methods to quantify the vectors, complexity of scaling-up, and quality control of these drugs also remain huge challenges. A typical 200 liters cGMP batch of AAV for a high-dose systemic delivery produces up to 200 doses for a cost approximating $2–3 million (Rajiv Gangurde, 2024; Williams, 2024). Despite tremendous efforts, progress is limited with often one or two log improvement in titers or vector potency (Yu et al., 2021; Viney et al., 2021; Zin et al., 2025). When considering the development costs of these drugs and the limited markets, the high-price tag (between 1 and 4 million a dose) becomes more understandable (Wong et al., 2023). Moreover, batch-to-batch variability, empty capsid removal, and purification efficiency are still challenging (Ebberink et al., 2022; Khaparde et al., 2025; Srivastava et al., 2021). Removal of empty capsids and other impurities may help reduce the immune activation (Li et al., 2022; McColl-Carboni et al., 2024). The lack of common standard for AAV characterization, potency assays, and quality control procedures represent another area needing improvement, but multiple efforts are ongoing to standardize the procedure (Yoganathan Jq and Quizi, 2025; Wang J. et al., 2024; Magnus and Kasbach, 2025).

There are also other challenges in the field to treat muscular dystrophies and neuropathies: timing of the procedures, endpoint and biomarker selection, immune challenges, long-term durability, and patient population heterogeneity (Lu et al., 2025; Di Minno et al., 2024; Shen et al., 2022). This last challenge represents a big hurdle for clinical trial enrollment (Stimpson et al., 2021). For muscular dystrophy in particular, the timing of the procedure is key. The affected muscles are progressively replaced by fibrofatty areas and therefore dilute the therapeutic effect over time as fibroblasts are poorly targeted by AAVs (Xie et al., 2024). The immune profile of each patient also needs to be defined before and during treatment to ensure that no preexisting immunity exists against the vector and that no reactions (TMA, enzyme elevations, and myositis) are detected against it or the transgene, particularly at higher doses (Manini et al., 2021; Mendell et al., 2024b). This is very important as, until now, immune reaction prevents a re-administration of the vector (Zambon et al., 2024).

## Converging modalities: CRISPR, epigenetic drugs, and next-generation strategies for neuromuscular gene therapy

In addition to gene replacement and exon-skipping strategies, several new approaches have emerged, such as clustered regularly interspaced short palindromic repeats (CRISPR)-based nucleases gene editing, base editing, prime editing and epigenome modulation (Adhikari et al., 2023; Qin et al., 2024) (Figure 1), and are now entering clinical development notably for SMA and DMD (Arbab et al., 2023; Stephenson et al., 2023; Young et al., 2016; Nicolau et al., 2025) and beyond [for recent reviews, see the studies by Lyu et al. (2025), O’Donnell et al. (2025), and Yuan et al. (2026)] (Figures 1, 2). All these techniques are certainly going to change the field by offering a high efficiency and precision with the promise of permanent or controlled changes. This is the case for CRISPR-based editing. CRISPR/cas9 allows permanent gene editing by replacing mutations in the DNA itself (Jinek et al., 2012), and CRISPR-based editors use a CRISPR-guided enzyme to precisely convert a cytosine to a thymine (cytosine base editor) or an adenine to a guanine (adenine base editor), both enabling single-base corrections without cutting the DNA (Figures 1, 2) (Komor et al., 2016). Multiple studies on human cells (Young et al., 2016; Li et al., 2015; Long et al., 2018; Min et al., 2019) and preclinical studies on mice (Young et al., 2016; Min et al., 2019; Long et al., 2014; Amoasii et al., 2017; Maino et al., 2021; Pickar-Oliver et al., 2021) and large animals (Amoasii et al., 2017; Oh et al., 2022; Yu et al., 2016; Zou et al., 2021; Chen et al., 2015) have demonstrated successful restoration of dystrophin expression using CRISPR-mediated exon-skipping and correction strategies, with newer genome editing tools such as base editors (Lin et al., 2024; Chai et al., 2023; Chemello et al., 2021) and prime editing (Chemello et al., 2021; Happi Mbakam et al., 2023; Wang Q. et al., 2024), offering increased specificity and reduced risk of double-stranded breaks. However, each technique has its own limitation and improvement trajectory: CRISPR-based nucleases remain unmatched for large, efficient genome modifications but suffer from issues linked to DNA double-strand breaks (DSBs) (p53 activation, complex chromosomal rearrangements, and deletions or loss) (Cullot et al., 2023; Tsuchida et al., 2023; Kosicki et al., 2018; Leibowitz et al., 2021) and off-target structural variants (Fu et al., 2013; Pacesa et al., 2022). Recent work focuses on optimizing fidelity (Fang et al., 2021; Lee et al., 2025; Kim et al., 2014; Fu et al., 2014), implementing mitigation strategies for a more controlled-size edits (Kosicki et al., 2022), controlling the lifetime of genome editors (Khajan et al., 2025), and offering alternative designs using artificial intelligence (Wang et al., 2019b) or synthetic molecules (Rahdar et al., 2015). Base editors offer highly efficient, DSB-free editing but are limited to specific context (single base edit) and show deaminase-linked off-targets (Komor et al., 2016). The current innovations focus on optimizing the deaminase domains and backbones to limit off-targets (Perrotta et al., 2025; Han et al., 2025), protospacer adjacent motif (PAM) (Zhang et al., 2020; Tan et al., 2020), and create conditional or transitory editing (Tong et al., 2023; Long et al., 2021), particularly for sensitive cells such as stem cells (Nandy et al., 2023; Escobar et al., 2021). Prime editors can generate larger edit with limited DSBs, despite a low efficiency and delivery (Anzalone et al., 2019). Despite the recent development of highly specific pegRNA design for prime editing (Liu et al., 2023; He et al., 2025), some challenges, namely, delivery, immunogenicity, and controlled off-target effects, remain [for a recent review, see the study by Chen and Liu (2023)]. Optimizing the delivery format (dual-AAV, nanoparticles, virus-like particles, and circular RNAs) of these different molecules is one of the current focus of the field (Yu et al., 2025; Chen et al., 2023; Holubowicz et al., 2025; Banskota et al., 2022; An et al., 2024; Zhang et al., 2025; Reautschnig et al., 2025). Despite current progress, many scientific and ethical challenges remain in understanding and improving immunogenicity, off-target effects, delivery, and efficacy, while also developing better scalable production methods (Holubowicz et al., 2025; Lazzarotto et al., 2026; Kim et al., 2020; Xu et al., 2024; Cetin et al., 2025).

Another promising approach is epigenetic modulation, which relies on modifications of DNA methylation, histone post-translational modifications, and chromatin architecture alterations to modify gene expression without changing the underlying genetic sequence [for recent reviews, see the studies by Consalvi et al. (2011) and Lunke and El-Osta (2009). These techniques, often relying on CRISPR-based technologies, offer several advantages over genome editing (no alteration of the underlying DNA sequence, fully tunable, and potentially reversible), particularly for dosage-sensitive genes (*SMN1/SMN2* for SMA) (Stigliano et al., 2025), diseases linked to sequence repeats [like CTG repeats in *DMPK* for Myotonic dystrophy type 1 (DM1) (Porquet et al., 2023), or the contraction of D4Z4 repeats in facioscapulohumeral muscular dystrophy (Das and Chadwick, 2021; Stavrou and Kleopa, 2023)]. Epigenic modulations can also be used to influence specific pathways such as inflammation or muscle damage (Bajanca and Vandel, 2017; Pigna et al., 2019). Yuan et al. (2026) provided a recent review recapitulating all the undergoing strategies. However, the control and durability of the epigenic changes, off-target chromatin modifications, and delivery issues particularly for large proteins are still hindering the applicability of these approaches (Pulecio et al., 2017). The field is working on chemically controlled recruitment systems (Chem-CRISPR/dCas9 platforms) (Altinbay et al., 2024) while also using large-scale drug screening, OMICS approaches, and AI-based design to identify new compounds and guide the specificity, longevity, and reversibility of epigenetic mediations for future therapeutic use (Louie et al., 2025; Salvati et al., 2025; Sabry et al., 2025). Of note, the first HDAC inhibitor drug, givinostat/Duvyzat, which modulates transcriptional programs to reduce inflammatory signaling, has been approved by the FDA for ambulant DMD patients aged ≥6 years (Lamb, 2024).

Pharmacological approaches aim at improving muscle function, reducing disease progression, or targeting specific molecular mechanism [for recent reviews, see the studies by Barthelemy and Wein (2018), Bartoli et al. (2023), Davalos and Kushlaf (2025), and Deng et al. (2022)], and are underway but are outside the scope of this article.

Finally, as AAVs are the most common gene therapy tool used in the field, several academic laboratories and biotechnology/pharmaceutical companies have engineered AAV capsids with better muscle tropism or nonviral platform to reduce the cost and avoid the undesired targeting of the liver. Several teams have engineered new capsids (e.g., LICA1 or MyoAAV) with impressive results (Eid et al., 2024; Ghauri et al., 2023; Nisanov et al., 2025; Vu Hong et al., 2024; Firnberg et al., 2025). Nonviral strategies have also been investigated (nanoparticles and muscle-homing peptides); however, despite promising preclinical data, they show very limited muscle penetration and diffusion (Colapicchioni et al., 2022; Huang et al., 2020; Li et al., 2025; Rao and Ganguli, 2024).

## Between uncertainty and precision: charting the future of neuromuscular gene therapy in the era of personalized medicine

Despite significant advancements in the past decade, the field is still very fragile. A lot of underexplored areas need significant advancements: tissue-specific immune modulation and interventions, pediatric-focused delivery, personalized genomics and biomarker selection, combination therapy, platform technology development, long-term monitoring and assessment, and health economic integration, among others (Bartoli et al., 2023; Landfeldt, 2022; Papadimas et al., 2021; Porcari et al., 2025).

The need for a more precise delivery, whether through the advancement of engineered AAV, nanoparticles, or any other technologies, coupled with the need for a pediatric-centric delivery system, is undoubtedly crucial (Stavrou et al., 2022; Kagiava and Kleopa, 2025; Liu et al., 2025). Most delivery systems are adapted from adult procedures, but pediatric muscle presents unique challenges, including tissue growth and changing vascular access, which will undoubtedly challenge the biodistribution and bioavailability of therapies (Shen et al., 2022; Manini et al., 2021). Technologies (growth-responsive vectors) or methods (staged delivery) that could sense and respond to these changes could be determinant and dramatically change the therapeutic landscape in the near future (Morgan and Muntoni, 2021).

To mitigate the immune risks associated with these approaches, multiple aspects are investigated: tissue-specific tolerance induction using tissue-specific promoters (Emami et al., 2023) and local delivery of immune suppressors or regulatory T-cell inducers (Emami et al., 2023; Seltrecht et al., 2024). Advancement in nonviral delivery could represent a good alternative as they show reduced immunogenicity and potential for re-dosing, but their efficiency is currently very low (Liu et al., 2025; Kenjo et al., 2021).

Current treatment guidance relies mainly on genetic mutations (Kazamel and Ho, 2023; Dabaj et al., 2024; Ng et al., 2022), but functional biomarkers are missing (Kariyawasam et al., 2019; Ba et al., 2021). The development of natural history databases (Balaji et al., 2025; Broomfield et al., 2023; Broomfield et al., 2024), comprehensive biomarker panels based on OMICS (Doorenweerd et al., 2017; Todd et al., 2025; Aydin et al., 2025), and imaging data (Xie et al., 2024; Todd et al., 2025; Senesac et al., 2020; Sherlock et al., 2021) could trigger precision medicine approaches, which will likely be the future era of the field.

Almost all the current strategies are focusing on the main affected tissue, but as any given treatment will allow the patient to live longer, additional organs will be impacted especially in case of a gene that is expressed in several tissues with some poorly targeted by current treatments like the heart or the brain. For example, although DMD is a neuromuscular disorder, there are several isoforms of this gene being expressed in the brain or liver (Doorenweerd et al., 2017; Tetorou et al., 2025; Garcia-Cruz et al., 2023). Thus, developing cardiac muscle-specific gene therapies or brain-targeted therapies, together with specific biomarkers and endpoints, could help mitigate these life-threatening aspects that will most likely become more prevalent with the emergence of patients who have received treatments that prolonged their life with unknown long-term effects.

Combination therapy is also likely going to be needed as single approach therapies are not sufficient to mitigate all aspects of some of these complex diseases (Barthelemy and Wein, 2018). Indeed, it is also important to keep in mind that combining gene therapy with anti-inflammatory agents (D’Ambrosio and Mendell, 2023; Saha et al., 2025), regenerative medicine and cell therapy (Escobar et al., 2025; Takenaka-Ninagawa et al., 2024; Pagliari et al., 2024), and immune (Spathis et al., 2025; Padhye et al., 2024) or epigenetic modulators (Aartsma-Rus, 2024; Mercuri et al., 2024) remains underexplored and will likely represent a new area of research in the future years.

Meanwhile, to accelerate therapeutic development, reduce development costs, and give a timely access to drugs to more patients, pharmaceutical companies are trying to develop platform technologies (FDA Guidance Documents, 2024) that share a common feature [same backbone chemistry for AON (Desjardins et al., 2022; Weeden et al., 2025) or same AAV serotype for gene delivery (Firnberg et al., 2025)] or common downstream pathways (membrane stability, inflammation, and fibrosis) (Dewi et al., 2025), promising an acceleration of therapy development for multiple related diseases or across multiple dystrophy types. Indeed, a platform technology designation bypasses the need to re-validate vector tropism, immune dynamics, or manufacturing for each new genetic target (Brooks et al., 2020).

Finally, the long-term monitoring and the need for a more sophisticated economic model are crucially needed (Wolff et al., 2025; Landfeldt, 2022). Current gene therapies are designed as a single treatment, but as these diseases are progressive and the long-term efficacy of these treatments is unknown, re-administration or therapeutic reinforcement may be needed over several years or decades (Wolff et al., 2025; Oskoui et al., 2025). Current guidance from the FDA mandates a 5- to 15-year follow-up for AAV therapies to monitor (transgene persistence, delayed adverse events, reproductive effects, or secondary malignancies) (FDA Guidance Documents, 2020). Consequently, developing immune tolerance protocols and engineered vectors or nonviral delivery methods, which enable safe redosing throughout a patient’s lifetime, is a necessity. The current US economic model seems unlikely to be sustainable for rare diseases where patients have limited time and access to alternative treatments as it focuses more on the drug costs than their global healthcare impact. As an example, DMD imposes per-patient annual societal costs between $80,120 and $120,910 across developed nations with indirect and informal caregiving costs constituting 18%–43% of total expenses (Landfeldt et al., 2014). A new model more adapted to the healthcare system impacts, relying on quality-adjusted life years, considering the caregiver burden, and that could support sustainable pricing and access (Postma et al., 2022; Nestler-Parr et al., 2018; Facey et al., 2021; Margaretos et al., 2022), is needed. Risk-sharing models (such as outcome-based pricing) (Facey et al., 2021; Margaretos et al., 2022) and global registries (such as California Neurodegenerative Disease Registry or Parent Project Muscular Dystrophy) (Gliklich Nad et al., 2014; CNDR, 2026; PPMD, 2023) may improve affordability and post-market surveillance.

Our analysis emphasizes that the field of gene therapy for neuromuscular dystrophies while having major breakthrough and promising results is at a critical junction. Success will likely require continued improvement in delivery systems, manufacturing processes, and immune modulation strategies. It will also require careful clinical trial design, increased monitoring, and more adaptive economic models, alongside a deeper understanding of disease biology and biomarker-guided personalization to improve treatment outcomes.
